# Letter to the editor: Left atrial enlargement in chronic kidney disease: A marker of risk or a target for intervention?

**DOI:** 10.1016/j.clinme.2026.100661

**Published:** 2026-10-15

**Authors:** Muhammad Nauman Hashmi, Pankaj Garg

**Affiliations:** King Abdullah Dialysis Centre, Ministry of National Guard Health Affairs, Jeddah, Saudi Arabia; King Abdullah International Medical Research Centre (KAIMRC), Saudi Arabia; University of East Anglia, Norwich, UK

Dear Editor,

We read with great interest the study by Suh *et al*. examining the prognostic significance of left atrial (LA) diameter in patients with chronic kidney disease (CKD).^1^ The investigators provide valuable long-term data from the KNOW-CKD cohort, demonstrating that LA enlargement was independently associated with kidney failure requiring renal replacement therapy and expanded major adverse cardiovascular events. These findings further highlight the close interaction between cardiac structural abnormalities and progressive kidney disease.

An important question is whether LA enlargement provides prognostic information beyond established CKD risk markers. LA enlargement reflects the cumulative effects of elevated left ventricular filling pressures and chronic diastolic loading. In CKD, these abnormalities frequently coexist with left ventricular hypertrophy, arterial stiffness, and volume overload, creating a pathophysiological substrate for heart failure and cardiovascular events.^2^ In patients with HFpEF, abnormalities of LA structure and function are also strongly related to disease severity and prognosis.^3^ Thus, LA enlargement may represent more than an isolated echocardiographic finding; it may serve as an integrated marker of chronic cardiovascular stress in CKD.

However, the use of LA diameter as the principal measure deserves consideration. LA geometry is three-dimensional, and a single linear diameter may not adequately capture atrial remodelling. Indexed LA volume and measures of LA function, including LA strain, may provide more comprehensive information regarding atrial remodelling and cardiovascular risk.^3^ Future CKD cohorts incorporating these parameters could determine whether they offer incremental prognostic value over conventional measures such as eGFR, albuminuria, left ventricular mass, and E/e′.

The findings also raise an important clinical question: should LA enlargement become part of routine cardiovascular risk stratification in CKD? The emerging cardiovascular-kidney-metabolic framework emphasises the interconnected nature of cardiac and renal disease and supports more integrated approaches to risk assessment.^4^ Nevertheless, prognostic association alone does not establish clinical utility. Prospective studies should determine whether identifying patients with LA enlargement leads to improved management or outcomes.

Finally, therapies that modify cardiovascular risk in CKD, including sodium-glucose cotransporter-2 inhibitors, may potentially influence the cardiac remodelling pathway, although whether such interventions specifically reverse LA enlargement or improve LA function in CKD remains uncertain.^5^

Overall, the study by Suh *et al*. strengthens the concept that the left atrium may provide an important window into the heart–kidney interaction. Future research integrating LA volume, strain, and longitudinal changes may help transform this echocardiographic marker from a prognostic association into a clinically actionable component of CKD risk assessment.

## CRediT authorship contribution statement

**Muhammad Nauman Hashmi:** Conceptualization, Data curation, Formal analysis, Writing – original draft, Writing – review & editing. **Pankaj Garg:** Conceptualization, Formal analysis, Writing – original draft, Writing – review & editing.

## Funding

This research did not receive any specific grant from funding agencies in the public, commercial, or not-for-profit sectors.

## Declaration of competing interest

The authors declare that they have no known competing financial interests or personal relationships that could have appeared to influence the work reported in this paper.
